# New data on *Wangaraneus* Mi, Wang & Li, 2024 (Araneae, Araneidae), with descriptions of two new species from Xizang, China

**DOI:** 10.3897/zookeys.1291.203690

**Published:** 2026-09-14

**Authors:** Huan Zhu, Cheng Wang, Guy Smagghe, Xiaoqi Mi

**Affiliations:** 1 School of Life and Health Science, Kaili University, Kaili 556011, Guizhou, China School of Life and Health Science, Kaili University Guizhou China; 2 College of Agriculture and Forestry Engineering and Planning, Guizhou Provincial Key Laboratory of Biodiversity Conservation and Utilization in the Fanjing Mountain Region, Tongren University, Tongren 554300, Guizhou, China College of Agriculture and Forestry Engineering and Planning, Guizhou Provincial Key Laboratory of Biodiversity Conservation and Utilization in the Fanjing Mountain Region, Tongren University Guizhou China; 3 Institute of Entomology, Guizhou University, Guiyang 550025, Guizhou, China Institute of Entomology, Guizhou University Guiyang China; 4 Department of Biology, Vrije Universiteit Brussel (VUB), 1050 Brussels, Belgium Department of Biology, Vrije Universiteit Brussel (VUB) Brussels Belgium

**Keywords:** Arachnida, Aranei, diagnoses, identification key, integrative taxonomy, new combination, new synonym, species delimitation, Tibet

## Abstract

New data on *Wangaraneus* Mi, Wang & Li, 2024 are provided based on morphological examination and mitochondrial cytochrome *c* oxidase subunit I (COI) sequencing. Two new species from Xizang, China, *W. delicatus* Mi & Wang, **sp. nov**. and *Wangaraneus fencha* Mi & Wang, **sp. nov**., are described and illustrated based on both sexes. In addition, two new combinations are proposed and established: *W. cruciformis* (Yin, Peng & Wang, 1994), **comb. nov**., transferred from *Singa* C.L. Koch, 1836, and *W. quadrituberculatus* (Roy, Sen, Saha & Raychaudhuri, 2014), **comb. nov**., transferred from *Chorizopes* O. Pickard-Cambridge, 1871. *Wangaraneus zioni* (Mi, Li & Pham, 2023), **syn. nov**. is synonymized with *W. cruciformis*. The newly described species are diagnosed based on detailed comparisons of somatic morphology and copulatory organs with similar congeneric species. Their placement within *Wangaraneus* is further supported by COI sequence evidence. An identification key to the known species (except *W. quadrituberculatus*) is provided.

## Introduction

*Wangaraneus* is a small genus containing only three species, which are mainly found in eastern Asia (WSC 2026). Of these, *W. ryani* (Mi, Li & Pham, 2023) is found in Vietnam; *W. yequ* Mi, Wang & Li, 2024 is found in southwestern China; and *W. zioni* (Mi, Li & Pham, 2023) is found in both Vietnam and southwestern China. All three species are known from both sexes (Mi et al. 2023, 2024). No revision work has been conducted on this small group yet. Similarly, no molecular studies have been conducted on this genus.

Two new species of *Wangaraneus* were recognized while examining materials from Xizang. Based on an examination of the type specimens and/or historical documents, two new combinations and one new synonym were also proposed.

## Materials and methods

The morphological examination and descriptive methods mainly follow those of Mi and Li (2022), while the molecular procedures follow the protocols described by Mi et al. (2024). All examined specimens are deposited in the Museum of Tongren University, China (TRU). Detailed information on the specimens used for molecular analyses, including voucher numbers, sexes, and GenBank accession numbers of partial mitochondrial cytochrome *c* oxidase subunit I (COI) sequences, is provided in Table 1.

**Table 1.** Voucher information and COI sequence data of specimens used for molecular analysis in the present study.

**Species**	**Voucher code**	**Sex**	**GenBank accession no**.
*Araneus guoi*	TRU-Araneidae-482/m-205	♂	PZ704238
*Araneus guoi*	TRU-Araneidae-483/m-206	♀	PZ704239
*Araneus luoi*	TRU-Araneidae-585/m-211	♂	PZ704240
*Araneus luoi*	TRU-Araneidae-586/m-212	♀	PZ704241
*Kongaraneus fani*	TRU-Araneidae-652/m-213	♂	PZ704242
*Kongaraneus fani*	TRU-Araneidae-653/m-214	♀	PZ704243
*Kongaraneus shilini*	TRU-Araneidae-688/m-215	♂	PZ704244
*Kongaraneus shilini*	TRU-Araneidae-689/m-216	♀	PZ704245
*Araneus cheni*	TRU-Araneidae-474/m-217	♂	PZ704246
*Araneus cheni*	TRU-Araneidae-475/m-218	♀	PZ704247
*Zangaraneus pani*	TRU-Araneidae-769/m-219	♂	PZ704248
*Zangaraneus pani*	TRU-Araneidae-770/m-220	♀	PZ704249
*Zangaraneus liui*	TRU-Araneidae-744/m-223	♂	PZ704250
*Zangaraneus liui*	TRU-Araneidae-745/m-224	♀	PZ704251
*Kongaraneus shenghangi*	TRU-Araneidae-676/m-225	♂	PZ704252
*Kongaraneus shenghangi*	TRU-Araneidae-677/m-226	♀	PZ704253
*Araneus jinhuai*	TRU-Araneidae-520/m-231	♂	PZ704254
*Araneus jinhuai*	TRU-Araneidae-521/m-232	♀	PZ704255
*Araneus lidiae*	TRU-Araneidae-548/m-233	♂	PZ704256
*Araneus lidiae*	TRU-Araneidae-549/m-234	♀	PZ704257
*Qianaraneus kejii*	TRU-Araneidae-708/m-235	♂	PZ704258
*Qianaraneus kejii*	TRU-Araneidae-709/m-236	♀	PZ704259
*Kongaraneus mengfeiae*	TRU-Araneidae-669/m-237	♂	PZ704260
*Kongaraneus mengfeiae*	TRU-Araneidae-670/m-238	♀	PZ704261
*Wangaraneus fencha* sp. nov.	TRU-Araneidae-811/m-279	♀	PZ704262
*Wangaraneus delicatus* sp. nov.	TRU-Araneidae-807/m-283	♀	PZ704263
*Wangaraneus delicatus* sp. nov.	TRU-Araneidae-808/m-284	♂	PZ704264
*Gea spinipes*	-	-	KJ957965.1

The COI sequences were used to assess genetic relationships among the taxa examined and to provide additional evidence for species identification and taxonomic placement. Phylogenetic reconstruction was performed using a neighbor-joining (NJ) approach implemented in MEGA 5.0 (Tamura et al. 2011), based on the Kimura 2-parameter (K2P) distance model. Node support was evaluated using 1000 bootstrap replicates. *Gea spinipes* C.L. Koch, 1843 was selected as the outgroup based on its taxonomic position within Araneidae.

All measurements are given in millimeters. Leg measurements are given as total length (femur, patella + tibia, metatarsus, tarsus). Abbreviations used in the text and figures are as follows: **ALE** anterior lateral eye; **AME** anterior median eye; **C** conductor; **CD** copulatory duct; **CO** copulatory opening; **DR** dorsal ramus; **E** embolus; **FD** fertilization duct; **MA** median apophysis; **MOA** median ocular area; **P** projection; **PLE** posterior lateral eye; **PME** posterior median eye; **VR** ventral ramus; **Sp** spermatheca; **TA** terminal apophysis.

## Results

**Figure 1.**
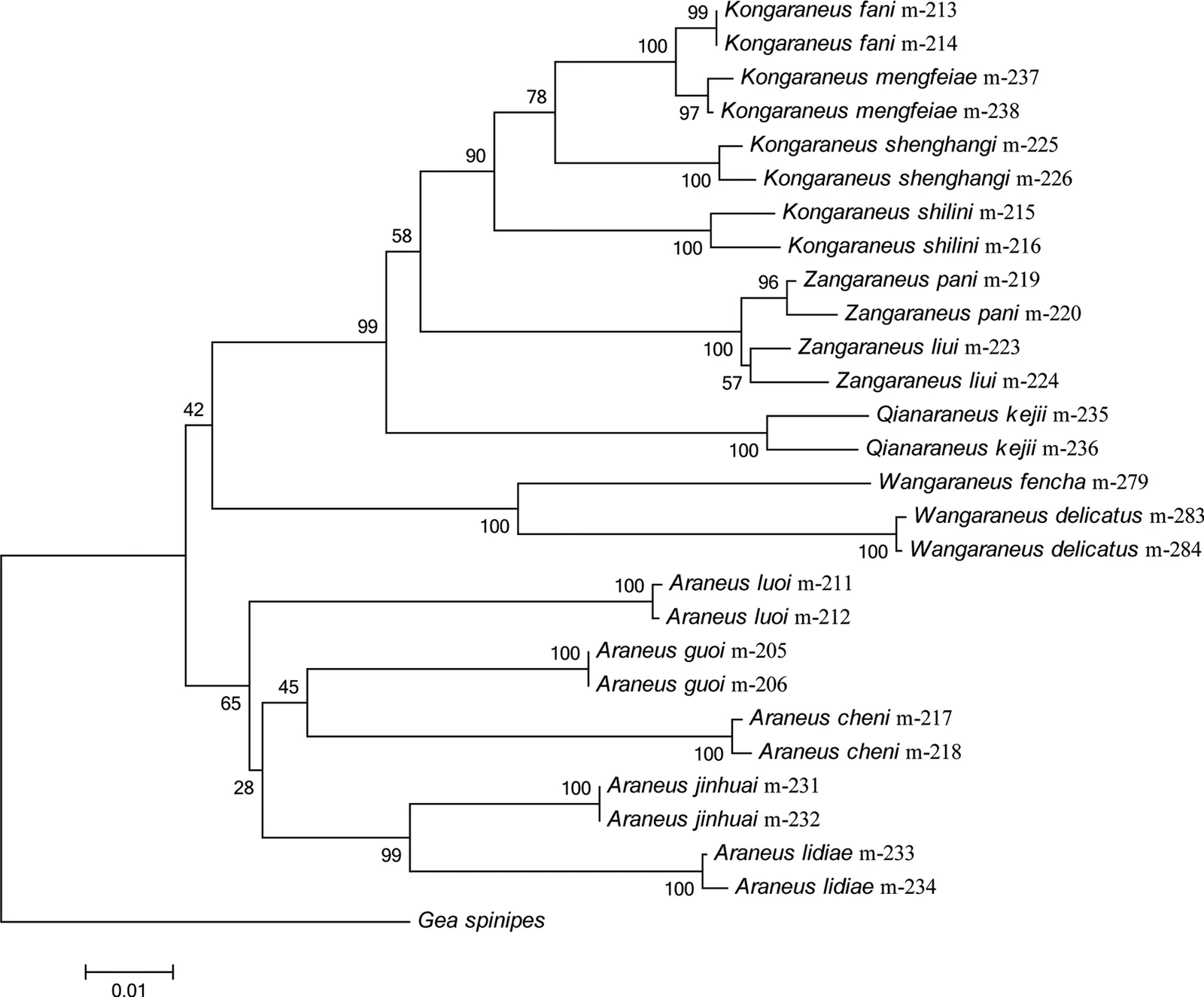
Neighbor-joining (NJ) tree generated using the K2P distance model based on COI sequences. Bootstrap values from 1000 replicates are shown at the nodes.

The specimens used for COI sequence analysis are listed in Table 1, including the outgroup *Gea spinipes* C.L. Koch, 1843 obtained from the National Center for Biotechnology Information. The neighbor-joining phylogenetic tree generated in this study is presented in Fig. 1. In the resulting tree, the two newly described species, *W. fencha* Mi & Wang, sp. nov. and *W. delicatus* Mi & Wang, sp. nov., clustered together in a strongly supported clade with a bootstrap value of 100%. This result is consistent with the morphological identification and provides additional molecular evidence supporting the placement of these two species within *Wangaraneus*.

### Taxonomy

**Family Araneidae Clerck, 1757**

**Genus *Wangaraneus* Mi, Wang & Li, 2024**

#### Wangaraneus

Taxon classificationAnimaliaAraneaeAraneidae

Mi, Wang & Li, 2024: 324.

9936A163-C15E-5710-94F4-149EF7077586

##### Type species.

*Wangaraneus yequ* Mi, Wang & Li, 2024 from Yunnan, China.

##### Diagnosis.

This genus can easily be distinguished from other genera in the family by its modified palpal tibia, which has one to five ctenidia on its dorsal ramus. It can also be distinguished from other genera by the abdomen being blunt anteriorly and truncated posteriorly, with pair of posterolateral humps.

##### Description.

Carapace longer than wide, pear-shaped, reflective; median ocular area wider posteriorly than anteriorly. Cervical groove conspicuous in females and inconspicuous in males; fovea somewhat longitudinal in males and depressed in females. Chelicerae with four promarginal and two retromarginal teeth. Sternum cordiform. Legs yellow with brown annuli; coxa I with hook, and femur II with corresponding groove, tibia II with several macrosetae. Abdomen longer than wide, blunt anteriorly and truncated posteriorly, with pair of posterolateral humps; dorsum with intermittent longitudinal paler bands and one or two transverse paler bands, reflective. Male palp with two patellar bristles; palpal tibia bifurcated, dorsal ramus always with ctenidia; cymbium covering most of bulb in prolateral view; paracymbium finger-like (except flattened and incised distally in *W. delicatus* sp. nov.); bulb elliptical; tegulum smooth, with low ridge between bases of median apophysis and conductor; median apophysis wide at base, somewhat flattened, lateral side approximately equal to edge of tegulum; embolus tapered or thread-like; conductor tongue-shaped or triangular; terminal apophysis slender and membranous or absent. Epigynal plate wider than long, convex or flat; copulatory openings located on posterior, lateral, or ventral surfaces; copulatory ducts approximately equal length to spermatheca, or even longer; spermathecae spherical or kidney-shaped, either touching each other or separated.

##### Composition.

*Wangaraneus ryani* (Mi, Li & Pham, 2023) from Vietnam, *W. yequ* Mi, Wang & Li, 2024 from China, *W. delicatus* Mi & Wang, sp. nov. from China, *W. fencha* Mi & Wang, sp. nov. from China, *W. cruciformis* (Yin, Peng & Wang, 1994), comb. nov. from China and Vietnam, and *W. quadrituberculatus* (Roy, Sen, Saha & Raychaudhuri, 2014), comb. nov. from India. All species are known by both sexes, but the male of *W. quadrituberculatus* has been misidentified.

##### Distribution.

China, India, Vietnam.

### Key to the species of *Wangaraneus*

Except *W. quadrituberculatus*, for which the origin of figures is unclear and the type specimens have not been examined.

**Table:** 

1	Female	**2**
–	Male	**6**
2	Epigynal plate with projection anteriorly (Fig. 7A–C)	***W. fencha* sp. nov**.
–	Epigynal plate without projection anteriorly	**3**
3	Copulatory openings situated on ventral surface (Mi et al. 2023: fig. 6A)	***W. ryani***
–	Copulatory openings situated on lateral or posterior surface	**4**
4	Copulatory openings situated on lateral surface (Mi et al. 2024: fig. 48C)	***W. yequ***
–	Copulatory openings situated on posterior surface	**5**
5	Epigyne round in posterior view (Fig. 4B)	***W. delicatus* sp. nov**.
–	Epigyne triangular in posterior view (Mi et al. 2024: fig. 51B)	***W. cruciformis***
6	Lacking terminal apophysis (Fig. 6)	***W. fencha* sp. nov**.
–	Terminal apophysis present	**7**
7	Dorsal ramus of palpal tibia with one ctenidium (Mi et al. 2024: fig. 47E)	***W. yequ***
–	Dorsal ramus of palpal tibia with 3–5 ctenidia	**8**
8	Conductor thick, somewhat triangular and arched (Fig. 3)	***W. delicatus* sp. nov**.
–	Conductor broad at base, tongue-shaped distally in apical view	**9**
9	Dorsal ramus of palpal tibia with 4 ctenidia (Mi et al. 2023: fig. 7A, B, E)	***W. ryani***
–	Dorsal ramus of palpal tibia with 5 ctenidia (Mi et al. 2024: fig. 50E)	***W. cruciformis***

#### Wangaraneus delicatus

Taxon classificationAnimaliaAraneaeAraneidae

Mi & Wang sp. nov.

D5F1E2FA-ABC3-5319-B06B-BF27FCE5BA6A

https://zoobank.org/19229B85-0CEB-4DE7-BD59-D4E0DE8696A1

**Figure 2.**
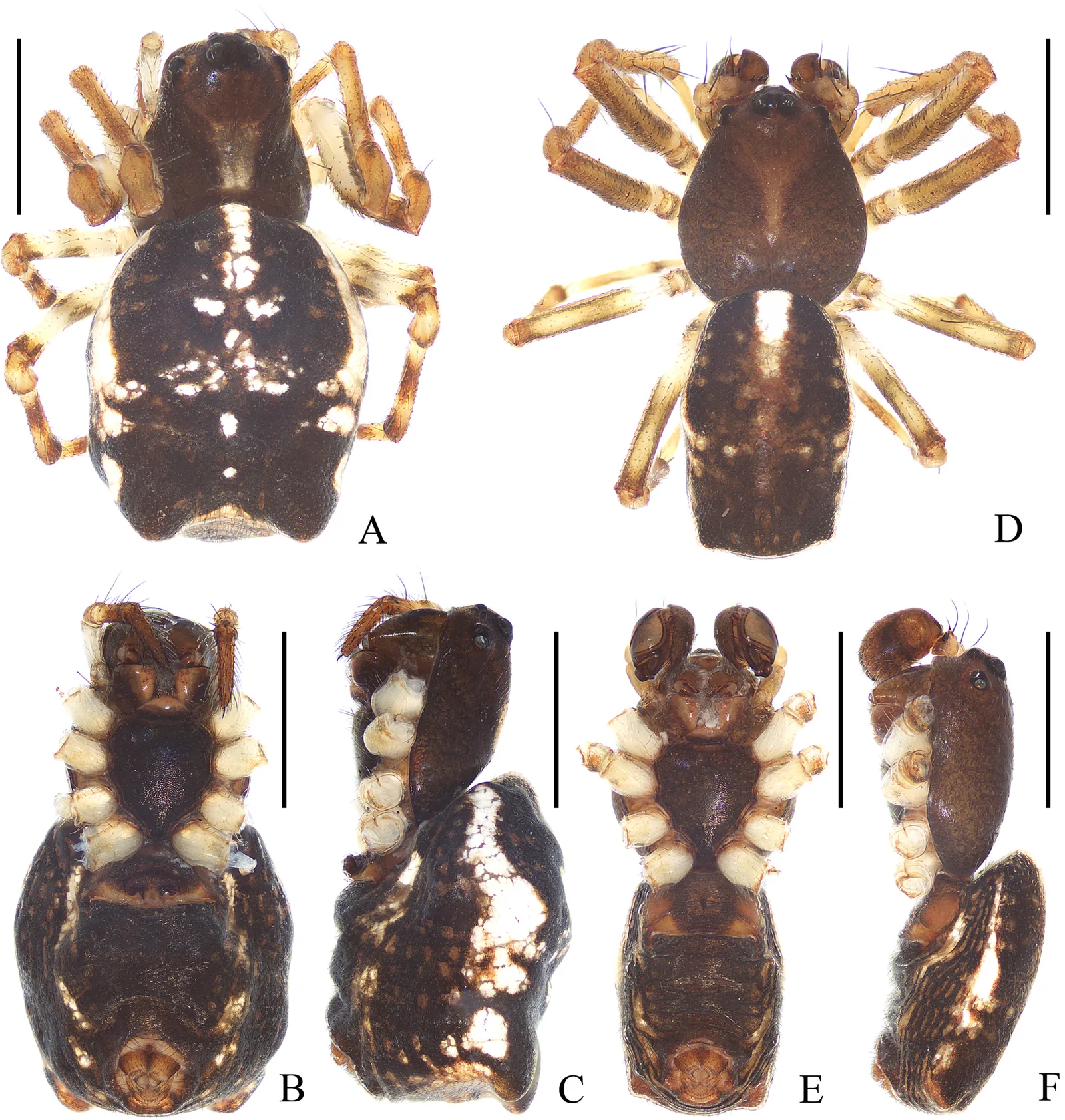
*Wangaraneus delicatus* Mi & Wang, sp. nov. **A–C**. Female paratype TRU-Araneidae-801; **D–F**. Holotype. **A, D**. Habitus, dorsal view; **B, E**. Ibid., ventral view; **C, F**. Ibid., lateral view. Scale bars: 1.0 mm. Photographs by X.Q. Mi.

**Figure 3.**
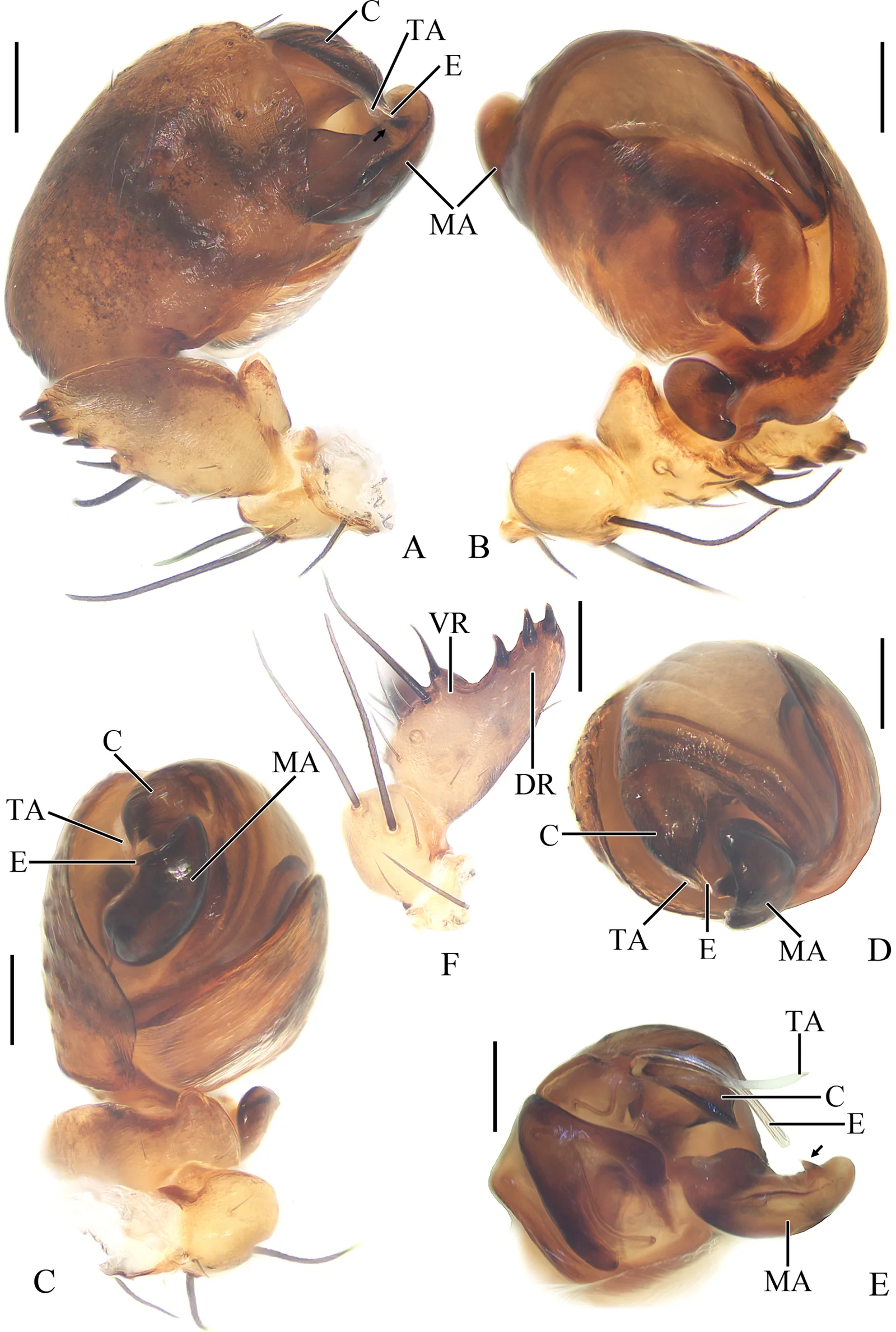
*Wangaraneus delicatus* Mi & Wang, sp. nov., holotype; **A**. Palp, prolateral view; **B**. Ibid., retrolateral view; **C**. Ibid., ventral view; **D**. Ibid., apical view; **E**. Expanded palp bulb; **F**. Patella and tibia of palp. Scale bars: 0.1 mm. Abbreviations: C, conductor; DR, dorsal ramus; E, embolus; MA, median apophysis; TA, terminal apophysis; VR, ventral ramus. Photographs by X.Q. Mi.

**Figure 4.**
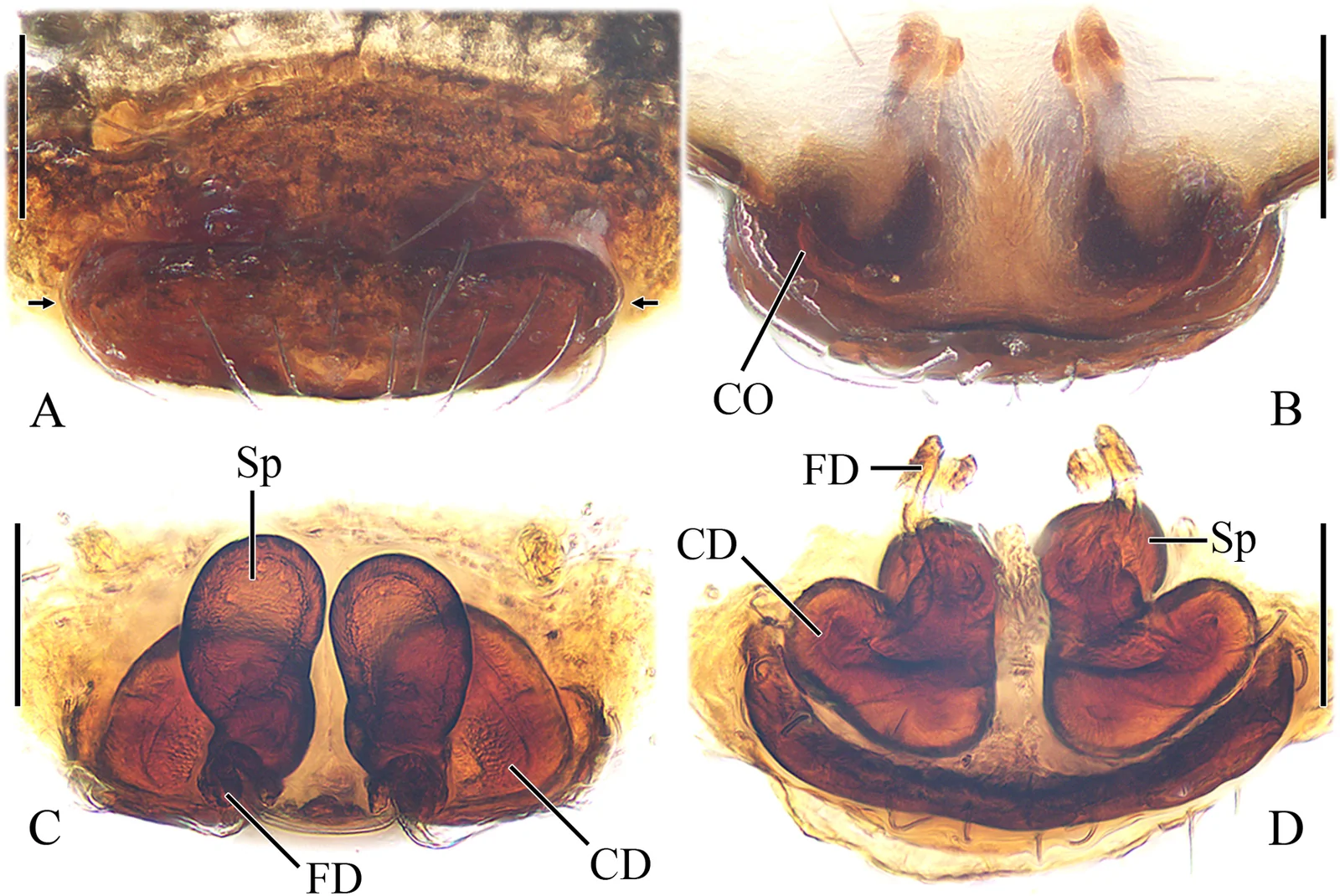
Female paratype TRU-Araneidae-801 of *Wangaraneus delicatus* Mi & Wang, sp. nov. **A**. Epigyne, ventral view; **B**. Ibid., posterior view; **C**. Vulva, dorsal view; **D**. Ibid., posterior view. Scale bars: 0.1 mm. Abbreviations: CD, copulatory duct; CO, copulatory opening; FD, fertilization duct; Sp, spermatheca. Photographs by X.Q. Mi.

Figs 2A–F, 3A–F, 4A–D

##### Chinese name.

柔嫩王园蛛.

##### Type material.

***Holotype***: China • ♂; Xizang Auton. Region: Dinggyê Co., Chentang Township, Natang Vill.; 27°50.47'N, 87°26.28'E, ca. 2390 m a.s.l.; 28 May 2025; C. Wang et al. leg.; TRU-Araneidae-800. ***Paratypes***: • 3♀♀1♂; same data as for holotype; TRU-Araneidae-801–804; • 1♀; same locality and collectors as for holotype; 22 May 2025; TRU-Araneidae-805; • 1♂; Chentang Township, Shalie Vill.; 27°52.43'N, 87°24.87'E, ca. 2390 m a.s.l.; 22 May 2025; C. Wang et al. leg.; TRU-Araneidae-806; • 1♀2♂♂; same locality and collectors as for holotype; 27 May 2025; TRU-Araneidae-807–809.

##### Diagnosis.

The new species resembles *W. yequ* in habitus and thread-like embolus, but can be distinguished as follows: 1) copulatory openings situated on posterior surface (Fig. 4B) vs lateral surface (Mi et al. 2024 fig. 48C); 2) spermathecae kidney-shaped, touching each other (Fig. 4C) vs spherical, separated (Mi et al. 2024 fig. 48D, E); 3) dorsal ramus of palpal tibia with three ctenidia (Fig. 3A, B, F) vs lacking ctenidia (Mi et al. 2024 fig. 47A, B, E); and 4) conductor heavily sclerotized, triangular (Fig. 3A, C–E) vs membranous, with sides nearly parallel (Mi et al. 2024 fig. 47A–D).

##### Description.

**Male** (holotype, Figs 2D–F, 3A–F). Total length 2.65. Carapace 1.30 long, 1.05 wide. Abdomen 1.60 long, 0.95 wide. Clypeus 0.15 high. Eye sizes and interdistances: AME 0.06, ALE 0.06, PME 0.10, PLE 0.06, AME-AME 0.10, AME-ALE 0.15, PME-PME 0.10, PME-PLE 0.18, MOA length 0.25, anterior width 0.20, posterior width 0.25. Leg measurements: I 4.65 (1.35, 1.60, 1.05, 0.65), II 4.40 (1.25, 1.55, 1.00, 0.60), III 3.05 (1.00, 1.00, 0.60, 0.45), IV 4.05 (1.30, 1.35, 0.90, 0.50). Carapace dark brown, paler in front of fovea. Chelicerae dark brown. Endites almost square, dark brown with paler inner tip. Sternum dark brown, approximately equal in length and width. Abdomen ~1.68× longer than wide, dark brown with longitudinal white band anteriorly and vaguely paler spots. Venter dark brown. Spinnerets yellowish brown.

***Palp*** (Fig. 3A–F): tibia weakly bifurcated, dorsal ramus with three ctenidia, ventral ramus with two ctenidia; paracymbium flattened and incised distally; median apophysis longer than wide, anti-clockwise curved in prolateral view, concave and with spur near distal end (see arrows in Fig. 3A, E); embolus thread-like; conductor thick, somewhat triangular and arched; terminal apophysis long, membranous.

**Female** (paratype TRU-Araneidae-801, Figs 2A–C, 4A–D). Total length 2.90. Carapace 1.30 long, 1.00 wide. Abdomen 1.95 long, 1.55 wide. Clypeus 0.10 high. Eye sizes and interdistances: AME 0.08, ALE 0.08, PME 0.13, PLE 0.08, AME-AME 0.08, AME-ALE 0.18, PME-PME 0.10, PME-PLE 0.20, MOA length 0.28, anterior width 0.23, posterior width 0.28. Leg measurements: I 4.00 (1.20, 1.40, 0.85, 0.55), II 3.60 (1.10, 1.25, 0.75, 0.50), III 2.60 (0.85, 0.85, 0.50, 0.40), IV 3.60 (1.20, 1.25, 0.70, 0.45). Habitus similar to that of male, but posterolateral humps on abdomen more obvious.

***Epigyne*** (Fig. 4A–D): epigynal plate flat anteriorly and transversally raised posteriorly, raised part (between arrows in Fig. 4A) ~3.7× wider than long in ventral view; copulatory openings arched, slit-shaped, situated on posterior surface; copulatory ducts approximately same width as spermatheca, bend upwards initially, then twist inwards and downwards to connect spermatheca at its ventral end; spermathecae kidney-shaped, with narrower ventral end, nearly touching each other at dorsal end.

##### Variation.

Total length: ♂♂ 2.65–3.10 (*N* = 5), ♀♀ 2.70–3.20 (*N* = 5).

##### Distribution.

China (only known from the type localities, Fig. 8).

##### Etymology.

The specific epithet is derived from the Latin word "*delicatus*", meaning delicate, referring to the membranous terminal apophysis; adjective.

##### Remarks.

Conspecificity of males and females has been supported by molecular evidence.

#### Wangaraneus fencha

Taxon classificationAnimaliaAraneaeAraneidae

Mi & Wang sp. nov.

27B500BB-7847-5391-99BE-D0FA4F7D68A7

https://zoobank.org/F0D88227-4CE4-4B57-8076-0BD5E00C783A

**Figure 5.**
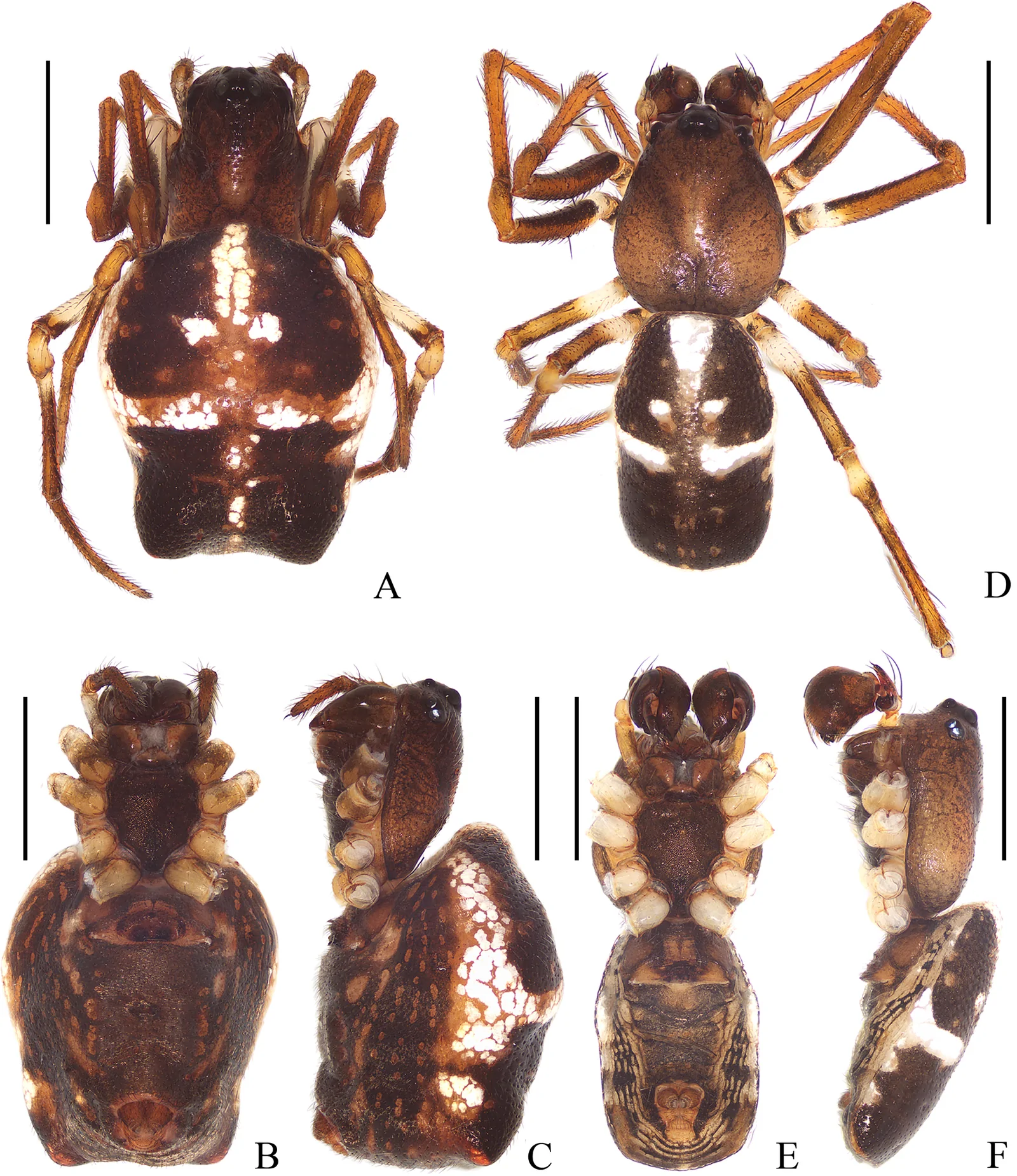
*Wangaraneus fencha* Mi & Wang, sp. nov. **A–C**. Female paratype TRU-Araneidae-811; **D–F**. Holotype. **A, D**. Habitus, dorsal view; **B, E**. Ibid., ventral view; **C, F**. Ibid., lateral view. Scale bars: 1.0 mm. Photographs by X.Q. Mi.

**Figure 6.**
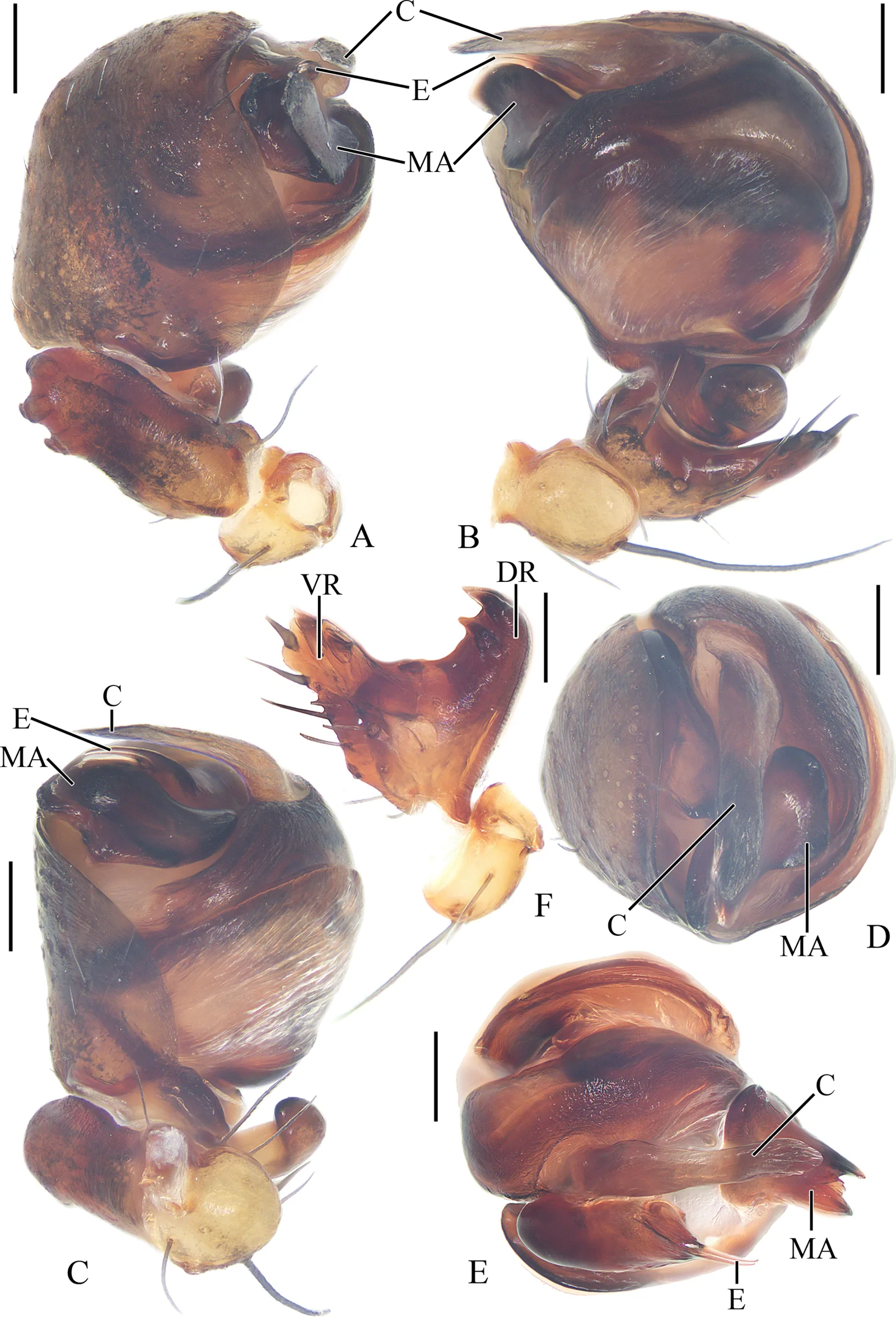
*Wangaraneus fencha* Mi & Wang, sp. nov., holotype; **A**. Palp, prolateral view; **B**. Ibid., retrolateral view; **C**. Ibid., ventral view; **D**. Ibid., apical view; **E**. Expanded palp bulb; **F**. Patella and tibia of palp. Scale bars: 0.1 mm. Abbreviations: C, conductor, DR dorsal ramus; E, embolus; MA, median apophysis; VR, ventral ramus. Photographs by X.Q. Mi.

**Figure 7.**
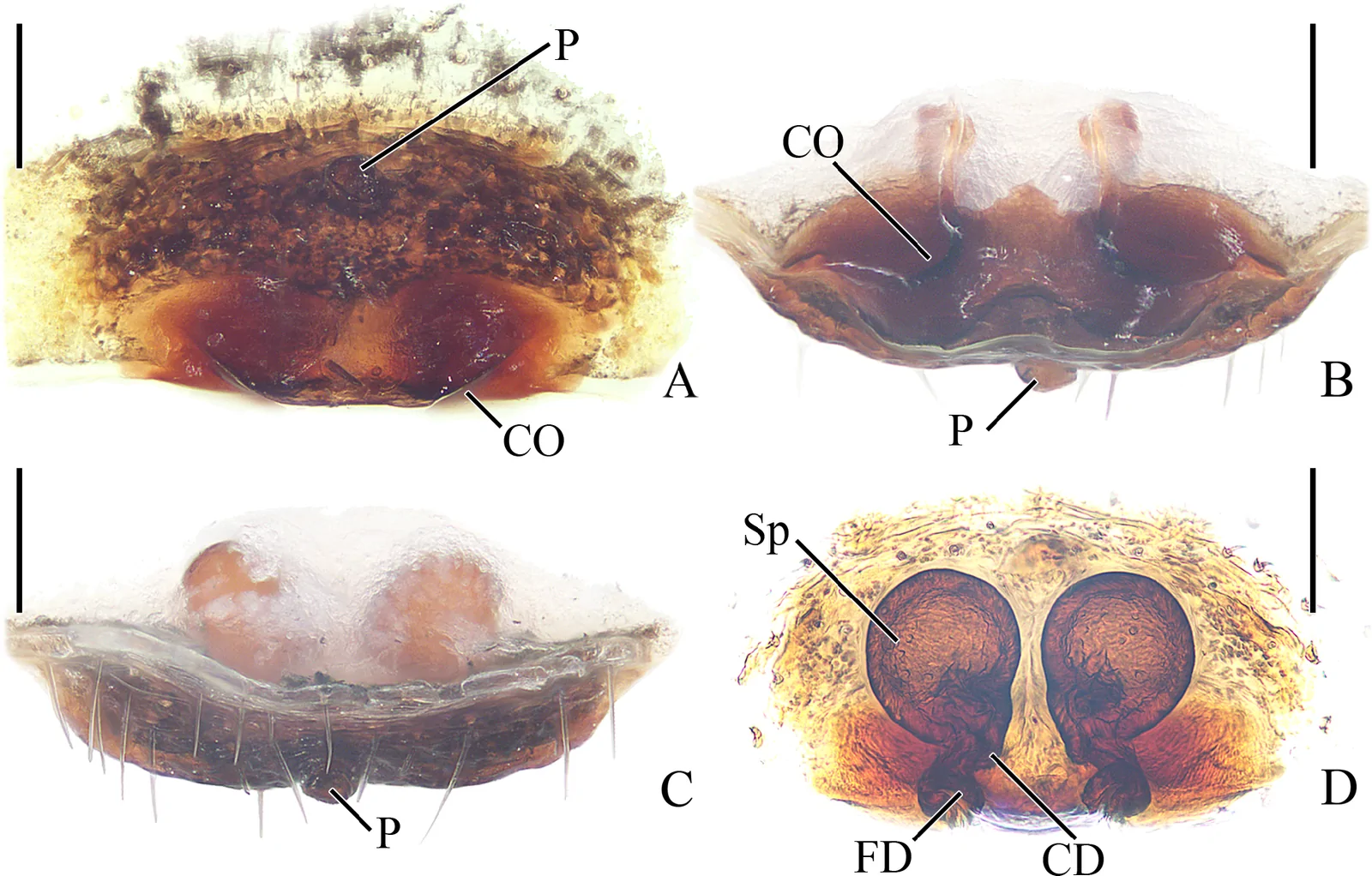
Female paratype TRU-Araneidae-811 of *Wangaraneus fencha* Mi & Wang, sp. nov. **A**. Epigyne, ventral view; **B**. Ibid., posterior view; **C**. Ibid., anterior view; **D**. Vulva, dorsal view. Scale bars: 0.1 mm. Abbreviations: CD, copulatory duct; CO, copulatory opening; FD, fertilization duct; P, projection; Sp, spermatheca. Photographs by X.Q. Mi.

Figs 5A–F, 6A–F, 7A–D

##### Chinese name.

分叉王园蛛.

##### Type material.

***Holotype***: China • ♂: Xizang Auton. Region, Linzhi City, Mêdog Co., Beibeng Township, Xirang Vill.; 29°10.78'N, 94°59.23'E, ca. 570 m a.s.l.; 16 Aug. 2023; X.Q. Mi et al. leg.; TRU-Araneidae-810. ***Paratypes***: • 6♀♀8♂♂; same data as for holotype; TRU-Araneidae-811–824; • 1♀1♂; Beibeng Township, Jiangxin Vill.; 29°13.70'N, 95°8.04'E, ca. 700 m a.s.l.; 17 Aug. 2023; X.Q. Mi et al. leg.; TRU-Araneidae-825–826; • 3♀♀3♂♂; Mêdog Co., Dexing Township, Yigongbai Vill.; 29°17.77'N, 95°11.32'E, ca. 1770 m a.s.l.; 18 Aug. 2023; X.Q. Mi et al. leg.; TRU-Araneidae-827–832; • 11♀♀10♂♂; Dexing Township, Deguo Vill.; 29°23.81'N, 95°22.35'E, ca. 800 m a.s.l.; 20 Aug. 2023; X.Q. Mi et al. leg.; TRU-Araneidae-833–853; • 1♀3♂♂; Mêdog Co., Mêdog Township, Mêdog Vill., around Renqingbeng Temple; 29°18.46'N, 95°21.49'E, ca. 1940 m a.s.l.; 18 Aug. 2023; C. Wang & H. Yao leg.; TRU-Araneidae-854–857; • 3♀♀3♂♂; Mêdog Township, Mêdog Vill., Haishishenlou Scenic Area; 29°20.62'N, 95°20.71'E, ca. 1290 m a.s.l.; 18 Aug. 2023; C. Wang et al. leg.; TRU-Araneidae-858–863; • 5♀♀3♂♂; Mêdog Co., Damu Township, roadside from Kabu Vill. to Damu Vill.; 29°28.70'N, 95°27.20'E, ca. 1380 m a.s.l.; 18 Aug. 2023; C. Wang & H. Yao leg.; TRU-Araneidae-864–871.

##### Diagnosis.

This species resembles *W. cruciformis* in habitus and deeply bifurcated palpal tibia, but can be distinguished as follows: 1) epigynal plate with projection anteriorly (Fig. 7A–C) vs lacking (Mi et al. 2024 fig. 51A–C); 2) posterior epigynal plate not raised (Fig. 7A–C) vs strongly raised (Mi et al. 2024 fig. 51A–C); 3) copulatory openings situated on sub-axial of posterior surface (Fig. 7B) vs situated laterally on posterior surface (Mi et al. 2024 fig. 51B); 4) spermathecae separated (Fig. 7D) vs touching each other (Mi et al. 2024 fig. 51D); 5) dorsal ramus of palpal tibia with three ctenidia (Fig. 6A, F) vs five ctenidia (Mi et al. 2024 fig. 50E); and 6) lacking terminal apophysis (Fig. 6A–E) vs terminal apophysis present (Mi et al. 2024 fig. 50A, C, D).

##### Description.

**Male** (Holotype, Figs 5D–F, 6A–F). Total length 2.90. Carapace 1.30 long, 1.05 wide. Abdomen 1.80 long, 1.00 wide. Clypeus 0.18 high. Eye sizes and interdistances: AME 0.06, ALE 0.06, PME 0.10, PLE 0.06, AME-AME 0.05, AME-ALE 0.10, PME-PME 0.08, PME-PLE 0.15, MOA length 0.20, anterior width 0.18, posterior width 0.23. Leg measurements: I 4.50 (1.35, 1.50, 1.05, 0.60), II 4.10 (1.25, 1.40, 0.90, 0.55), III 2.80 (0.95, 0.90, 0.55, 0.40), IV 3.90 (1.25, 1.30, 0.85, 0.50). Carapace yellowish brown. Chelicerae dark brown. Endites almost square, dark brown with paler inner tips. Sternum dark brown, approximately equal in length and width. Legs yellowish brown with paler annuli on femora II, III, and IV. Abdomen ~1.8× longer than wide, dark brown with white patch anteriorly and two pairs of white patches at middle. Venter dark brown. Spinnerets yellowish gray.

***Palp*** (Fig. 6A–F): tibia deeply bifurcated, dorsal ramus with three ctenidia, ventral ramus with two ctenidia; median apophysis wide at base, bifurcated and pointed to tip of cymbium distally; embolus thick at base, tapered into fine tip; conductor membranous, with sides nearly parallel, about 4.67× longer than wide in apical view.

**Female** (paratype TRU-Araneidae-811, Figs 5A–C, 7A–D). Total length 3.00. Carapace 1.35 long, 0.95 wide. Abdomen 2.15 long, 1.65 wide. Clypeus 0.15 high. Eye sizes and interdistances: AME 0.06, ALE 0.06, PME 0.10, PLE 0.06, AME-AME 0.08, AME-ALE 0.13, PME-PME 0.10, PME-PLE 0.15, MOA length 0.25, anterior width 0.20, posterior width 0.28. Leg measurements: I 3.90 (1.20, 1.35, 0.85, 0.50), II 3.45 (1.05, 1.20, 0.75, 0.45), III 2.40 (0.80, 0.80, 0.45, 0.35), IV 3.70 (1.15, 1.25, 0.80, 0.50). Habitus similar to that of male, but posterolateral humps on abdomen more obvious.

***Epigyne*** (Fig. 7A–D): epigynal plate ~ 2× wider than long in ventral view, with small, round projection anteriorly, posterior half heavily sclerotized, forming inverted isosceles trapezoid; copulatory openings deeply concave, situated on posterior surface; copulatory ducts approximately equal length to spermathecal diameter, and connected spermatheca at its posterior surface; spermathecae spherical, less than their radius apart.

##### Variation.

Total length: ♂♂ 2.60–2.90 (*N* = 32), ♀♀ 2.50–3.00 (*N* = 30).

##### Distribution.

**Figure 8.**
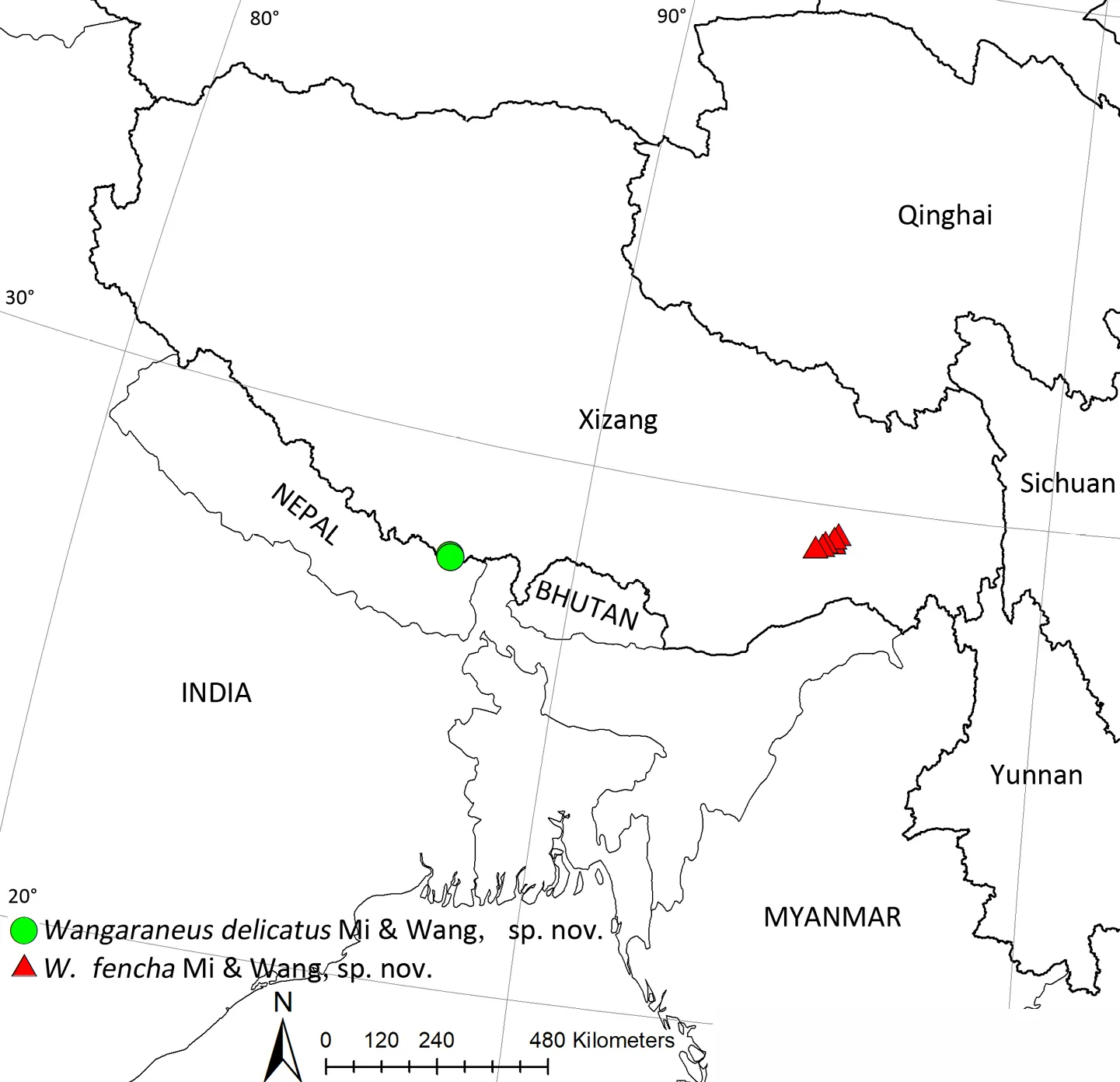
Distribution of the newly described species of *Wangaraneus* in China.

China (only known from the type localities, Fig. 8).

##### Etymology.

This species is derived from the Chinese pinyin "*fencha*", meaning bifurcated, in reference to the distally bifurcated median apophysis; adjective.

##### Remarks.

Male paratype TRU-Araneidae-825 and female paratype TRU-Araneidae-826 were collected on the same web.

#### Wangaraneus cruciformis

Taxon classificationAnimaliaAraneaeAraneidae

(Yin, Peng & Wang, 1994) comb. nov.

3ABB1642-2FC8-54E6-A687-5E8950B088F5

Singa cruciformis Yin, Peng & Wang, 1994: 109, figs 27–31 (♀♂); Yin et al. 1997: 392, fig. 284a–e (♀♂); Song et al. 1999: 309, figs 183G–H, O–P, 185K (♀♂). (holotype, ♀, allotype, ♂, paratype, 2♀, China: Yunnan Prov., Mengla Co., 12 Aug. 1983, J. Wang leg., deposited in the Museum of Hunan Normal University, Changsha, examined).
Hypsosinga zioni Mi, Li & Pham, 2023: 83, figs 8A–G, 10E–H (♀). (holotype, ♀, Vietnam: Cao Bang Prov., Sac Ha Vil., 17 July 2000, D.-S. Pham leg., deposited in the Institute of Zoology, Chinese Academy of Sciences, Beijing, examined). syn. nov.
Wangaraneus zioni : Mi et al. 2024: 326, figs 49A–F, 50A–E, 51A–D (♀♂).

##### Distribution.

China (Yunnan), Vietnam (Cao Bang).

##### Comment.

*Wangaraneus cruciformis* (Yin, Peng & Wang, 1994), comb. nov. is transferred from *Singa* to *Wangaraneus* based on the habitus and male palp similarity to the generotype *W. yequ*. Examination of the type material reveals that *S. cruciformis* is conspecific with *W. zioni*. Therefore, *H. zioni* Mi, Li & Pham, 2023 is here synonymized with *W. cruciformis* (Yin, Peng & Wang, 1994).

#### Wangaraneus quadrituberculatus

Taxon classificationAnimaliaAraneaeAraneidae

(Roy, Sen, Saha & Raychaudhuri, 2014) comb. nov.

04776808-4D09-52F2-9D5E-BAD3294EC795

Chorizopes quadrituberculata Roy et al., 2014: 4, figs 1–5, 12 (♀); Sen et al. 2015: 117, figs 723–727, pl. 23 (♀). Type material not examined.

##### Distribution.

India (West Bengal).

##### Comment.

*Chorizopes quadrituberculata* (Roy, Sen, Saha & Raychaudhuri, 2014) is transferred to *Wangaraneus* based on the resemblance of the habitus to the generotype. Though figures of the male palp (Roy et al. 2014: figs 9–11) of *C. quadrituberculata* are not clear enough for identification, judging from the male habitus (Roy et al. 2014: figs 6, 13), it must belong to the genus *Gasteracantha* Sundevall, 1833.

## Supplementary Material

XML Treatment for Wangaraneus

XML Treatment for Wangaraneus delicatus

XML Treatment for Wangaraneus fencha

XML Treatment for Wangaraneus cruciformis

XML Treatment for Wangaraneus quadrituberculatus

## Additional information

### Artificial Intelligence (AI) use

The authors accept full responsibility for the content of the manuscript, including the disclosure of any use of AI.

No AI tools were used in the preparation of this manuscript.

### Author ORCIDs

C. Wang https://orcid.org/0000-0003-1831-0579

G. Smagghe https://orcid.org/0000-0001-8334-3313

X. Mi https://orcid.org/0000-0003-1744-3855

### Data availability

All of the data that support the findings of this study are available in the main text.

## References

[B1] Clerck C (1757) Aranei Svecici. Svenska spindlar, uti sina hufvud-slågter indelte samt under några och sextio särskildte arter beskrefne och med illuminerade figurer uplyste. Laurentius Salvius, Stockholmiae [= Stockholm], 154 pp. 10.5962/bhl.title.119890

[B2] Mi XQ, Li SQ (2022) On eleven new species of the orb-weaver spider genus *Araneus* Clerck, 1757 (Araneae, Araneidae) from Xishuangbanna, Yunnan, China. ZooKeys 1137: 75–108. 10.3897/zookeys.1137.96306 PMC9836476 36760478

[B3] Mi XQ, Li SQ, Pham DS (2023) On five new species of the genera *Araneus* and *Hypsosinga* (Araneae, Araneidae) from Vietnam. ZooKeys 1161: 69–87. 10.3897/zookeys.1161.102375 PMC10206528 37234740

[B4] Mi XQ, Wang C, Li SQ (2024) Description of six new genera and twenty species of the orb-weaver spider family Araneidae (Araneae, Araneoidea) from Xishuangbanna, Yunnan, China. Zoological Research: Diversity and Conservation 1(4): 290–341. 10.24272/j.issn.2097-3772.2024.023

[B5] Roy TK, Sen S, Saha S, Raychaudhuri D (2014) A new *Chorizopes* O.P.-Cambridge, 1870 (Araneae: Araneidae) from West Bengal, India. Romanian Journal of Biology – Zoology 59(1): 3–9.

[B6] Sen S, Dhali DC, Saha S, Raychaudhuri D (2015) Spiders (Araneae: Arachnida) of reserve forests of Dooars: Gorumara National Park, Chapramari Wildlife Sanctuary and Mahananda Wildlife Sanctuary. World Scientific News 20: 1–339.

[B7] Song DX, Zhu MS, Chen J (1999) The Spiders of China. Hebei Science and Technology Publishing House, Shijiazhuang, 640 pp.

[B8] Tamura K, Peterson D, Peterson N, Stecher G, Nei M, Kumar S (2011) MEGA5: Molecular Evolutionary Genetics Analysis using maximum likelihood, evolutionary distance, and maximum parsimony methods. Molecular Biology and Evolution 28(10): 2731–2739. 10.1093/molbev/msr121 PMC3203626 21546353

[B9] WSC [World Spider Catalog] (2026) World Spider Catalog. Version 27. Natural History Museum Bern. [accessed on 1 July 2026.] 10.24436/2

[B10] Yin CM, Peng XJ, Wang JF (1994) Seven new species of Araneidae from China (Arachnida: Araneae). Acta Arachnologica Sinica 3: 104–112. [in Chinese]

[B11] Yin CM, Wang JF, Zhu MS, Xie LP, Peng XJ, Bao YH (1997) Fauna Sinica: Arachnida: Araneae: Araneidae. Science Press, Beijing, 460 pp. [in Chinese]

